# The double-edged sword effect of illegitimate tasks on hotel industry employees’ self-control behavior

**DOI:** 10.3389/fpsyg.2026.1785362

**Published:** 2026-04-28

**Authors:** Wenjing Ke, Bingjie Sun

**Affiliations:** 1 College of Business, Wuyi University, Wuyishan, China; 2 School of Business Administration, Xinjiang University of Science & Technology, Korla, China

**Keywords:** double-edged sword effect, employee self-control behavior, illegitimate tasks, power distance, work autonomy

## Abstract

**Introduction:**

Illegitimate tasks are a significant workplace stressor in the hotel industry, yet their impact on employees’ self-control behavior remains inconclusive, with prior studies reporting both negative and positive effects.

**Methods:**

Drawing on conservation of resources (COR) theory and the job demands-control model, this study examines the nonlinear impact of illegitimate tasks on self-control behavior, with power distance as a mediator and work autonomy as a moderator, using two-wave survey data from 536 hotel employees in China.

**Results:**

Illegitimate tasks exhibit a U-shaped relationship with self-control behavior and an inverted U-shaped relationship with power distance. Power distance mediates the nonlinear link between illegitimate tasks and self-control, and perceived work autonomy moderates (steepens) the inverted U-shaped relationship between illegitimate tasks and power distance.

**Discussion:**

These findings reconcile contradictory prior results by revealing the double-edged, nonlinear nature of illegitimate tasks, and provide practical guidance for hotel managers on task allocation, autonomy design, and employee support systems.

## 1 Introduction

The double-edged sword effect of illegitimate tasks on employees’ self-control behavior in the hotel industry has garnered increasing attention (Rasool et al., 2024). Employees’ self-control is crucial in determining hotel service quality (Mann and Ward, 2007; Baumeister, 2018). Although illegitimate tasks are a significant potential stressor, their impact on work performance has often been overlooked (Harari et al., 2021). Illegitimate tasks are assignments that exceed employees’ job responsibilities, should not be handled by them, or are inconsistent with their roles (Fan et al., 2023). For example, front desk staff may handle financial reimbursements or housekeeping staff may manage security duties note that such tasks breach employees’ psychological contracts, thus undermining job satisfaction. Prior research has mainly addressed the negative effects of illegitimate tasks (Ouyang et al., 2022; Dong et al., 2025). Ding and Kuvaas (2023) argue that these tasks lead to emotional exhaustion (Koch and Adler, 2018), work alienation, and role conflict. They also weaken organizational commitment and work engagement (Dong and Zhang, 2022), and further report adverse outcomes such as reduced self-control (Kim et al., 2019; Kottwitz et al., 2025). However, recent research explores possible positive outcomes. Found that illegitimate tasks may drive proactive learning. Most survey samples come from traditional industries such as manufacturing, IT, education, and government, with high psychological resilience. As a result, the literature presents divergent views on the impact of illegitimate tasks. Most survey samples come from traditional industries such as manufacturing, IT, education, and government. The hotel industry remains underexplored (Zeng et al., 2021). This gap leaves key questions unanswered. For example, do hotel employees have specific reactions to illegitimate tasks? How do these tasks influence their self-control (De Ridder et al., 2012)?

Compared to manufacturing employees, hotel workers perform more emotional labor and need greater self-control (Muraven and Baumeister, 2000). These increased emotional and regulatory demands may make them more sensitive to illegitimate tasks. As a result, such tasks are more likely to lead to role conflict and unfairness. This can then cause emotional exhaustion and alienation. Despite these risks, it is important to note that when illegitimate tasks align with professional norms, they can also boost self-efficacy, engagement, and self-control (Mann and Ward, 2007). Therefore, the impact of illegitimate tasks on hotel employees can be nonlinear, with both positive and negative outcomes possible. According to the conservation of resources (COR) theory (Hobfoll, 2001; Hobfoll et al., 2018), illegitimate tasks may act as challenges that promote coping. However, they can also be hindrances that cause burnout. This complexity is increased because personal resources can further shape how organizational support affects outcomes (Farh et al., 2007). As illegitimate tasks intensify, employees’ perceptions and self-control vary. This confirms the double-edged sword analogy (Logue, 1988).

To deeply analyze the mechanism of illegitimate tasks, this paper uses COR theory as its main framework. In employment relationships, individuals want to protect resources and gain new ones (Hobfoll et al., 2016). When leaders assign tasks seen as illegitimate, these demands threaten employees’ resources. Such tasks breach psychological contracts (Deng et al., 2018), create role conflict, and generate unfairness. However, finishing planned work can help workers regain resources. It shows compliance with norms and respect for leaders. As a result, leader trust and perceived support grow (Hobfoll et al., 2018). When given illegitimate tasks, employees start a cognitive appraisal process. They compare leader authority with the need to protect personal resources (Westman et al., 2004). As illegitimate tasks increase, the balance between duty and expected resources breaks down. This affects self-control. Work autonomy also shapes the nonlinear tie between illegitimate tasks and self-control (Ni et al., 2023). People differ in how they see and value resources. Hotel employees may react emotionally in various ways, even to the same illegitimate task (Zeng et al., 2021). In summary, this paper explores the link between illegitimate tasks and self-control behaviors of hotel workers using COR theory. Power distance mediates this relationship, and work autonomy is a moderating factor. These are used to explore effects on self-control (Nelson and Craighead, 1977). The key contributions are as follows. First, by using a nonlinear view, this study links conflicting research findings. It offers another way to understand illegitimate tasks. Second, by examining power distance, the study explains the effects of illegitimate tasks. This extends research on their connection to power distance. Third, the study finds boundary conditions, adding to the literature on individual work autonomy in hotels. Pindek et al. (2024) introduced illegitimate tasks as stressors. Dysvik and Kuvaas (2013) said illegitimate tasks are unreasonable or unnecessary. Unreasonable tasks go beyond role expectations or skills. Unnecessary tasks have no valid reason or value. Illegitimate tasks break psychological contracts or fairness. They signal disrespect or belittling. Such work challenges professional identity. Such tasks bring psychological stress (Nebioglu et al., 2012; Nasarullah et al., 2025). Illegitimate tasks drain self-control and cause exhaustion (Halbesleben et al. 2014; Sonnentag and Lischetzke, 2018). This results in fatigue and frustration. How employees see these tasks depends on resources and the work environment. These factors shape their perceptions (Tapola and Niemivirta, 2008). Therefore, the impact of illegitimate tasks varies depending on individual differences (Muraven and Baumeister, 2000) and the work environment (Tapola and Niemivirta, 2008).

Noted that employees assess whether work tasks align with their professional standards. They categorize these tasks as either compliant or illegitimate. Compliant tasks have a positive impact on employee well-being and organizational effectiveness. Illegitimate tasks produce adverse effects. Building on this, the conservation of resources (COR) theory (Tripathi, 2025; Ye et al., 2022), as outlined by Hobfoll (2001) and Hobfoll et al. (2018), provides a crucial framework for understanding the stress–strain process. In this process, resource loss refers to the consumption and depletion of personal resources, as with illegitimate tasks. Resource conservation relates to employees’ efforts to acquire, maintain, and protect their resources. When job demands do not match employees’ resource reserves, illegitimate tasks are perceived as threats to resource loss (Demerouti, 2025). A situation where resource loss exceeds resource gain is called a resource loss spiral. Drawing on these principles, most research has explored the negative consequences of illegitimate tasks (Javed et al., 2024). However, most work has viewed illegitimate tasks as either negative or positive, focusing only on single effects. This approach overlooks complex, nonlinear relationships from dynamic resource fluctuations over time (Javed et al., 2019). The literature has also neglected the unique impact of illegitimate tasks on hotel employees’ emotional resources and professional identity. This paper addresses that gap.

Given the high emotional investment and role ambiguity inherent in hotel work, hotel industry employees often face low-demand, non-compliant task requirements that fall below their expected role scope, as they struggle to cope with such demands effectively (Kaplan and Maehr, 2006). These tasks are interpreted as challenges in the workplace; completing them not only demonstrates employees’ adaptability and professional qualities but may also bring recognition and growth, thus prompting hotel industry employees to view them as opportunities to enhance their personal abilities and value (Westman et al., 2004). In summary, based on the Conservation of Resources-Loss of Resources model, low levels of illegitimate tasks may be perceived by hotel industry employees as challenging stressors, which can enhance their sense of achievement and efficacy, stimulate work engagement and positive emotions, thereby reducing their self-control behaviors (Mann and Ward, 2007). However, when non-compliant task requirements are excessively high and exceed the personal resources and capacity reserves of hotel industry employees, even with great effort and psychological energy, they find it challenging to achieve the expected goals (Senko et al., 2011). At the same time, excessive illegitimate tasks will continue to consume the psychological and cognitive resources of hotel industry employees, forcing them to engage in more self-regulation, which, in turn, leads to accelerated resource depletion (Halbesleben et al., 2014). In addition, excessive illegitimate tasks will trigger defensive psychology among hotel industry employees and elicit negative emotional responses such as anger and anxiety (Zhao et al., 2023). Therefore, based on the Conservation of Resources (COR) theory, excessively high requirements for illegitimate tasks will lead to excessive loss of employee resources and a sharp increase in psychological pressure, thereby increasing self-control behaviors among hotel industry employees (Halbesleben et al., 2014). Based on the above analysis, this study proposes the following hypothesis:

*H1*: The impact of illegitimate tasks on employees’ self-control behavior in the hotel industry is nonlinear. As the level of illegitimate tasks perceived by employees increases, their self-control behavior first decreases and then increases, exhibiting a U-shaped relationship.

### 1.1 The mediating role of power distance

In the previous research, power distance was primarily conceptualized as a cultural dimension distinguishing between ‘high power distance’ and ‘low power distance’ cultures (Bochner and Hesketh, 1994). Individuals in high power distance cultures exhibit greater acceptance of power inequalities within organizations (Huang et al., 2003), thereby identifying with and upholding hierarchical systems and top-down authority. In contrast, individuals in low power-distance cultures expect more egalitarian relationships (Daniels and Greguras, 2014). However, dichotomizing power distance solely along this cultural dimension limits a comprehensive understanding of its multilevel impact mechanisms. Subsequent research has demonstrated that the psychological contract established between organizations and individuals involves complex cognitive and behavioral processes (Basabe and Ros, 2005; Kirkman et al., 2009). Although investigations into power distance have progressively deepened from macro-level cultural concepts to measurable individual traits and dynamic psychological perceptions (Hochwarter et al., 2023), its mediating role in specific work contexts remains insufficiently understood. Integrating existing research with the present theme, power distance can be defined as the degree to which employees accept and expect an unequal distribution of power within an organization (Matusitz and Musambira, 2013; Tripathi, 2025). This conceptualization focuses on individuals’ internal cognition, emphasizing that when superiors make decisions or issue instructions to subordinates, such authoritative behavior is enacted through subordinates’ psychological identification, which helps maintain the organizational hierarchy (Montani et al., 2020). When employees face illegitimate tasks or unconventional work requirements assigned by superiors, a high power distance orientation interacts with and is activated by employees’ tendency to obey authority (Dong et al., 2023).

In the hotel industry, which places strong emphasis on service standards and hierarchical management, employees’ behavior patterns and psychological expectations are closely tied to the organizational structure (Lampaki et al., 2025). Hotel employees in high power distance settings often accept instructions more readily, comply with arrangements, and recognize and uphold leadership authority (Wang and Xie, 2023). To gain organizational recognition and advance their careers, hotel industry employees tend to demonstrate compliance and initiative, delivering efficient execution and service performance that improves the quality of interactions with superiors (Matta et al., 2015). When faced with low-level, illegitimate tasks, hotel industry employees can effectively respond to and complete them by utilizing their professional qualities and adaptability (Grant and Berry, 2011). At this point, from a conservation-of-resources perspective, employees view moderate illegitimate tasks as opportunities to showcase their personal value, representing a resource investment that may yield future returns (Lampaki et al., 2025). Successfully handling such tasks demonstrates employees’ loyalty and flexibility, helping them gain more recognition and trust from their superiors and acquire additional resources. Therefore, moderate illegitimate tasks promote positive interactions between employees and leaders, there by enhancing the acceptance of power distance (Montag et al., 2016). However, when illegitimate tasks become excessive and exceed the capabilities and resource reserves of hotel industry employees, the resource investment required begins to outweigh potential returns (Park and Gong, 2022). The employees’ initial positive responses and expectations become frustrated, and, due to the imbalance between resource investment and return, hotel industry employees cannot obtain the recognition and rewards they deserve for their additional efforts (Dobrev et al., 2006).

Furthermore, excessive demands from illegitimate tasks lead employees to feel unfairly treated and experience emotional exhaustion, prompting them to question the rationality of organizational functions and the authority of leaders (Desyllas et al., 2022). In this context, hotel industry employees perceive a serious mismatch between their efforts and rewards (Montani et al., 2020). Consequently, employees tend to reduce their proactive investment and emotional commitment, while leaders increasingly rely on positional power to exert pressure. Excessive illegitimate tasks undermine the trust foundation between superiors and subordinates, leading to a decline in the effectiveness and acceptance of power distance (Yan et al., 2020). In summary, as the intensity of illegitimate tasks increases, the power distance between leaders and employees first rises and then falls, forming an inverted U-shaped relationship that reflects the dynamic interplay between resource investment and resource depletion (Farh et al., 2007).

Research has confirmed that the nature of the relationship between leaders and employees, whether characterized by direct influence or by an interactive exchange process involving both structural and psychological elements, significantly affects employees’ self-control behaviors (Gong et al., 2009). First, in the hotel industry, a moderate power distance helps establish role clarity and behavioral norms among employees. A reasonable power distance contributes to higher psychological empowerment among employees, promotes better resource allocation, enhances their role identification and organizational commitment, and reduces the need for self-control behaviors (Hochwarter et al., 2023). From a resource conservation standpoint, when employees operate within a well-defined power distance that provides clear expectations and support, they experience less resource depletion from ambiguity and conflict, thereby conserving psychological resources (Montani et al., 2020). Second, given that service work in the hotel industry requires high emotional labor from employees, a well-functioning power distance can promote greater procedural justice, which, in turn, helps buffer employees’ perceptions of role stress and reduces inner conflict (Maslyn et al., 2017). In such circumstances, employees engage in their work with a more positive and proactive attitude, enhancing their internal identification with organizational norms, and their self-control behaviors decrease significantly as fewer resources are required for emotional regulation (Grewal et al., 2013). Ultimately, in the hotel industry’s service-oriented approach centered on customer centrality, the quality of interpersonal interaction (Xia et al., 2023). Between employees and their leaders, in addition to formal work contracts, a social exchange based on mutual respect operates (Luan et al., 2025). In relationships characterized by high-quality power distance, employees’ perceptions of leader support and organizational trust are enhanced, providing them with valuable resources (Matta et al., 2015). This psychological perception significantly increases employees’ acceptance of organizational requirements and their willingness to fulfill them, reducing the resource expenditure associated with resistance or self-regulation (Chin et al., 2025). Therefore, as power distance quality improves and develops in a benign manner, employees’ self-control behaviors will decrease.

In summary, illegitimate tasks, as a typical work stressor, represent a concentrated manifestation of the mismatch between organizational demands and individual resources, role conflict, and psychological contract breach (Dobrev et al., 2006). Employees passively accept illegitimate tasks, and to cope with such extra-role requirements, they need to consume additional psychological resources, undergoing a process of cognitive evaluation and emotional regulation (Kim et al., 2017). However, at different levels of illegitimate tasks, the power distance between leaders and employees undergoes dynamic changes driven by resource considerations (Kim et al., 2019). The higher the pressure on illegitimate tasks, the more pronounced the trajectory of changes in power distance, with an initial increase followed by a decrease as resource investment gives way to resource depletion (Ouyang et al., 2022). Meanwhile, the power distance relationship negatively influences employees’ self-control behaviors by either providing resources that reduce the need for self-regulation or consuming resources that necessitate greater self-control (Jiang et al., 2024). Therefore, power distance plays a mediating role in the nonlinear relationship between illegitimate tasks and self-control behaviors, transmitting the effects of fluctuating resource dynamics (Yuan and Zhou, 2015). Based on this, the following hypotheses are proposed:

*H2a*: There is a nonlinear relationship between illegitimate tasks and power distance. As employees perceive a higher level of illegitimate tasks, power distance initially rises and then falls, forming an inverted U-shaped relationship.

*H2b*: Power distance negatively influences self-control behavior.

*H2c*: Power distance mediates the nonlinear relationship between illegitimate tasks and self-control behavior.

### 1.2 The moderating role of work autonomy

Work autonomy refers to the extent to which employees perceive that they have control over the initiation, process, and outcomes of their work, beyond the constraints set by the organization (Tisu et al., 2023). This sense of control leads employees to exhibit different psychological and behavioral responses, reflecting perceptions of job characteristics (Dong et al., 2023; Roznowski and Wontorczyk, 2024). Employees with greater work autonomy have a greater capacity to cope with work challenges, as they have greater decision-making authority and freedom of action; consequently, they are more likely to proactively internalize and integrate their job responsibilities, demonstrating higher levels of organizational citizenship behavior (Lee et al., 2025). However, some studies suggest that work autonomy may exacerbate role stress when employees face ambiguous or conflicting tasks, as the relatively free decision-making space can lead to role ambiguity, negative work experiences, and adverse psychological states (Peng and Lee, 2019). Additionally, work autonomy may lead employees to perceive that the organization has abandoned necessary supervision and control over work processes, thereby damaging their organizational identification and reducing their acceptance of leadership authority (Kalsoom et al., 2024). Aubé et al. (2007) pointed out that employees’ perceived control over the work environment and their degree of autonomy in work tasks are closely related to their work attitudes and behaviors. Therefore, job autonomy affects employees’ cognitive evaluation and behavioral responses to illegitimate tasks. Due to the industry’s particularities and job requirements, many young employees enter the hotel service industry, and, with their age and pursuit of individuality and self-worth, job autonomy has become a core work characteristic that hotel employees particularly value (Bao et al., 2021). Thus, job autonomy is a boundary condition that influences hotel employees’ reactions to illegitimate tasks (Luan et al., 2025). Compared to employees with lower perceived work autonomy, those with high work autonomy typically exhibit stronger self-control; they can more effectively mobilize resources to cope with challenges and thus gain a greater sense of achievement and psychological empowerment (Ke et al., 2025). When faced with low to moderate levels of illegitimate task pressure, their high level of self-control enables them to respond with more flexible strategies and a more positive mindset (Zhao et al., 2020). From the perspective of resource conservation, this resource-gaining process is perceived by employees as an investment, which stimulates proactive behaviors, prompting them to complete illegitimate tasks to demonstrate their capabilities and value, thereby deepening interactions and trust with leaders and establishing high-quality power-distance relationships (Al-Homayan, 2013). However, when employees with high work autonomy face excessively high illegitimate task demands, these demands suddenly increase their resource consumption and psychological pressure, leading to intense feelings of frustration and questioning of leadership decisions (Zhang et al., 2017). This accelerated resource loss breaches the psychological contract and rapidly deteriorates the quality of power distance relationships. Therefore, when employees perceive a higher level of work autonomy, the relationship curve between illegitimate tasks and power distance exhibits a relatively steep inverted U-shape, as subsequent resource losses quickly offset the initial resource gains once task illegitimacy reaches a critical threshold (Alaydi and Ng, 2024). In contrast, for employees with lower perceived work autonomy, the change in power distance quality is relatively gradual as the level of illegitimate tasks increases, because their more limited resource reserves and coping capacity result in less pronounced resource fluctuations (Zeng et al., 2021). Based on the above analysis, grounded in conservation resources theory, which explains how the dynamic interplay between resource gain and loss generates nonlinear outcomes, we propose the following hypothesis:

*H3*: Perceived work autonomy plays a moderating role in the inverted U-shaped relationship between illegitimate tasks and power distance. When employees have a higher perceived level of work autonomy, the inverted U-shaped relationship between illegitimate tasks and power distance becomes steeper; when they have a lower perceived level of work autonomy, it is flatter.

**Figure 1:**
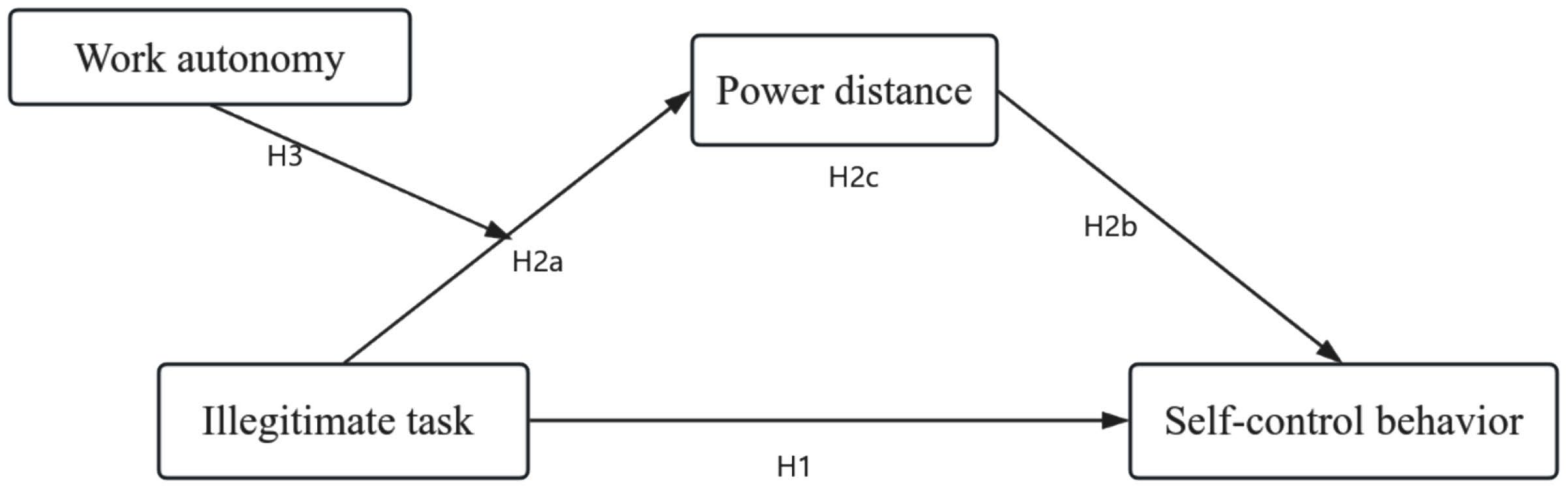
Research model.

In conclusion, the research model presented in this paper is illustrated in Figure 1.

## 2 Research design

### 2.1 Questionnaire design

This study collected data through a questionnaire survey that included basic demographic information (gender, age, educational background, and work experience) and four core variable scales measuring illegitimate task behavior, power distance, perceived work autonomy, and self-control behavior. The measurement of these variables drew on established scales from both domestic and international research. Specifically, illegitimate tasks refer to work assignments that exceed employees’ job responsibilities, should not be undertaken by them, or are inconsistent with their duties (Fan et al., 2023). This scale was developed by Semmer et al. (2010), subsequently refined, and finally finalized with eight items. Power distance refers to the degree to which employees accept and endorse unequal power distribution in organizations (Matusitz and Musambira, 2013). The six-item scale from Dorfman and Howell (1988) was adopted. Perceived work autonomy reflects the extent of control employees have over their work pace, methods, and decisions (Thompson and Prottas, 2006). The six-item scale developed by Thompson and Prottas (2006) was used. Self-control behavior in this study is operationally defined as the frequency with which employees actively inhibit negative emotions, restrain impulsive reactions, and persist in completing in-role tasks when facing illegitimate tasks in the workplace. It is neither a stable personality trait (e.g., general trait self-control), nor a state of ego depletion (i.e., temporary resource exhaustion), nor a service constraint (e.g., surface acting in emotional labor). Instead, it is conceptualized as situationally driven, active self-regulation behavior, encompassing task persistence, interpersonal restraint, and cognitive reappraisal, consistent with the nine-item scale developed by Nebioglu et al. (2012). All scales were developed using the standard back-translation method: items were first translated from English to Chinese and then back-translated into English to ensure accuracy (Shan et al., 2023). To align the measurement context with China’s hotel industry, this study made contextual adjustments based on the operational characteristics of domestic hotels and employees’ work conventions, thereby optimizing the scales. To avoid central tendency response bias, following the approach of Fan et al. (2023), all variables employed a 6-point Likert scale (1 = “strongly disagree,” 6 = “strongly agree”).

### 2.2 Data collection

To reduce common method bias, this study used a two-stage questionnaire survey targeting in-service, full-time frontline staff as well as participants enrolled in the “Starry Sky” training program, which is a specialized talent development initiative by the Education Branch of the China Hotel Association with the goal of “cultivating backbone personnel, enhancing skills, and promoting employment” through theoretical learning and on-the-job practice. Since its launch in 2018, over 5,000 hotel industry professionals have benefited from this program, including front desk attendants, housekeeping staff, catering and banquet service staff, concierges, shift managers, supervisors, deputy department managers, and other hotel professionals. A total of 536 valid responses were obtained from this sample. It is important to note that the “Starry Sky” program is a selective, career-advancement initiative, and its participants—who voluntarily enroll and are often screened—tend to exhibit stronger career development motivation, higher skill levels, and greater training engagement compared to the general frontline workforce. This introduces a selection bias: the sample overrepresents employees with proactive attitudes and potentially higher self-control resources, while underrepresenting those with lower motivation or higher burnout. Consequently, the findings derived from this sample may have limited generalizability to the broader population of frontline hotel employees, especially those who are less career-oriented or more disengaged. Future research should adopt random sampling across the industry to validate and extend these results.

The research team completed the questionnaire survey in two steps. The first-phase questionnaire primarily included demographic variables such as gender, age, educational level, and work experience, as well as the participants’ employee numbers and initials of their Chinese names (which were encoded for use only in matching data from both phases). Additionally, it included scales measuring illegitimate tasks and power distance. From September to October 2024, the survey was distributed through the hotel’s internal training system, where the participants worked. A total of 1,021 questionnaires were recovered in the first phase, with a recovery rate of 85.1%. The second-phase questionnaire included perceived work autonomy, demographic variables (also encoded to match data from both phases), self-control behavior, and the power distance scale. After completing the first-phase questionnaire recovery, the research team sent the second-phase questionnaire links to participants who had completed the first phase between November and December 2024. A total of 886 questionnaires were recovered in the second phase, with a recovery rate of 86.8%. All survey data were strictly confidential and used solely for this purpose.

After the questionnaires were collected, this study matched the two sets using the respondents’ enrollment year and student ID. After removing questionnaires with incomplete answers, obviously contradictory answers, patterned responses, Obvious social desirability, or extreme values, a total of 536 valid questionnaires were finally obtained. Among the valid samples, 42.5% are male and 57.5% are female; 68.3% are aged 18–30, 24.6% are aged 31–40, 6.7% are aged 41–55, and 0.4% are aged 55 and above; 8.2% have junior high school education or below, 32.6% have high school education, and 59.2% have bachelor’s degrees; in terms of years of work experience, 25.7% have less than 1 year, 48.1% have 2–5 years, 12.3% have 5–8 years, and 13.9% have more than 8 years.

## 3 Research results

### 3.1 Common method bias test

To reduce common method bias, this study employed procedural control methods, including multi-stage questionnaire collection, protection of respondent anonymity, and balancing item order during the survey phase. Additionally, this study employed Harman’s single-factor test to assess common method bias. In an unrotated exploratory factor analysis, four factors with eigenvalues greater than 1 were identified, and the first factor accounted for 26.5% of the total variance, which is below the critical threshold of 40%. The results indicate that there is no serious standard method bias.

### 3.2 Reliability and validity analysis

This paper utilizes a coefficient to assess the reliability of the variables. As shown in Table 1, the coefficients for illegitimate tasks, power distance, self-control behavior, and work autonomy are 0.922, 0.912, 0.889, and 0.903, respectively, all exceeding the 0.7 threshold. These results indicate that the items of each subscale have high internal consistency and strong measurement reliability. This study used AMOS 26.0 to conduct confirmatory factor analysis (CFA) on the sample. The initial CFA results showed that, except for item LMX5 (which had a factor loading below 0.5), all other items had factor loadings greater than 0.5. Following Fornell and Larcker’s recommendations, we removed LMX5 and reran the CFA. The revised CFA showed that all remaining items had factor loadings above 0.5. As presented in Table 2, the four-factor model (*χ*^2^/df = 3.767, CFI = 0.925, TLI = 0.901, RMSEA = 0.083) demonstrated a significantly better fit than competing models, indicating good discriminant validity. As shown in Table 1, the average variance extracted (AVE) for each factor exceeds 0.5, and composite reliability (CR) exceeds 0.7, confirming that all variables possess good convergent validity. Moreover, the square root of the AVE for each factor is substantially larger than its correlations with other factors, further supporting discriminant validity.

**Table 1:** Confirmatory factor analysis and reliability and validity indicators.

Variables	Items	Factor loading	AVE	CR	Cronbach is a
Illegitimate tasks, IT	IT1	0.815	0.675	0.945	0.947
	IT2	0.832			
	IT3	0.836			
	IT4	0.856			
	IT5	0.843			
	IT6	0.855			
	IT7	0.843			
	IT8	0.836			
Power distance PD	PD1	0.853	0.634	0.915	0.908
	PD2	0.846			
	PD3	0.786			
	PD4	0.732			
	PD5	0.778			
	PD6	0.756			
	PD7	0.786			
Self-control behavior SCB	SCB1	0.767	0.691	0.899	0.899
	SCB2	0.824			
	SCB3	0.857			
	SCB4	0.863			
work autonomyWA	WA1	0.734	0.641	0.905	0.903
	WA2	0.805			
	WA3	0.822			
	WA4	0.832			

**Table 2:** Model fitting index.

Model	*χ* ^2^	*df*	*χ*^2^/*df*	RMSEA	CFI	TLI
Four factors model (IT, PD, WA, SCB)	1235.235	322	3.978	0.081	0.901	0.911
Three factors model (IT + PD, WA, SCB)	3023.521	325	9.423	0.134	0.745	0.678
Two factors model (IT + PD + WA, SCB)	4402.653	327	14.422	0.157	0.576	0.575
Single factor model (IT + PD + WA + SCB)	5432.353	328	16.654	0.179	0.457	0.423

Clarification of “self-control behavior” and explanation for a potentially low mean score. In this study, “self-control behavior” is operationally defined as the frequency with which employees actively inhibit negative emotions, restrain impulsive reactions, and persist in completing in-role tasks when confronted with illegitimate tasks in the workplace. It is neither a stable personality trait (e.g., general trait self-control), nor a state of ego depletion (i.e., temporary resource exhaustion), nor a service constraint (e.g., surface acting in emotional labor). Instead, it reflects situationally driven active regulation behavior that encompasses task persistence, interpersonal restraint, and cognitive reappraisal. If the data reveal a low mean score for self-control behavior (e.g., below the theoretical midpoint of the scale), plausible reasons include: (1) frequent illegitimate tasks continuously deplete employees’ self-control resources, thereby reducing their willingness and ability to exert active self-control; (2) in a high power-distance culture, employees tend to adopt passive obedience rather than active regulation, which suppresses genuine self-control behavior; (3) many scale items involve curbing negative reactions (e.g., “I can refrain from losing my temper”), which is difficult to sustain under high pressure and perceived unfairness; (4) the occupational characteristics of the sample (e.g., frontline service workers) already entail high levels of emotional labor, and additional illegitimate tasks further compress the space for self-control. Thus, a low mean score indicates that illegitimate tasks and high power distance weaken employees’ self-control, consistent with theoretical expectations.

### 3.3 Correlation analysis

A correlation analysis of all variables yielded correlation coefficients (Table 3). The results show that illegitimate tasks and power distance, illegitimate tasks and self-control behavior, and power distance and self-control behavior are significant. There is a significant correlation between them, providing a basis for the next step in hypothesis testing.

**Table 3:** Correlation coefficient of main variables.

Variable	Mean	SD	1	2	3	4
Illegitimate tasks	3.787	1.091	1.000			
Power distance	4.421	0.765	−0.114*	1.000		
Self-control behavior	2.943	0.905	0.235	−0.187	1.000	
work autonomy	3.374	1.124	0.567	−0.086	0.396	1.000

*Respectively, indicate significant correlation at the *p* < 0.01 and *p* < 0.05 levels.

### 3.4 Hypothesis testing

This paper presents four regression models developed through least-squares regression and tested for statistical significance. To reduce the impact of multidisciplinary, improve model readability, and enhance numerical stability, all variables in this paper are subjected to centralization processing. Model 1 treats power distance as the dependent variable, illegitimate tasks and their square term as independent variables, and includes control variables such as gender, age, and tenure. Model 2, based on Model 1, adds the moderating variable job autonomy, as well as interaction terms between job autonomy and illegitimate tasks and the square term of illegitimate tasks. Model 3 constructs a regression model with work autonomy behavior as the dependent variable, illegitimate tasks, and the square term of illegitimate tasks as independent variables, along with control variables. Model 4, based on Model 3, adds the mediating variable power distance. The regression results are shown in Table 4.

**Table 4:** Summary of regression results.

Variables	Model 1 (Power distance)				Model 2 (Self-control)				Model 3 (Self-control)				Model 4 (Self-control)			
	Coef	SE	*p*	VIF	Coef	SE	*p*	VIF	Coef	SE	*p*	VIF	Coef	SE	*p*	VIF
Illegitimate tasks	1.059*	0.318	0.001	2.31	1.403*	0.421	0.001	2.45	−0.335	0.280	0.232	2.28	−0.189	0.245	0.440	2.35
Illegitimate tasks square	−1.198*	0.360	0.001	2.55	−1.354*	0.406	0.001	2.67	0.579*	0.174	0.001	2.50	0.409	0.218	0.061	2.58
work autonomy					−0.025	0.038	0.512	1.18								
					0.525*	0.262	0.045	1.22								
					−0.495*	0.247	0.045	1.20								
Power distance (m)													−0.145	0.056	0.010	1.35
Gender	0.234*	0.070	0.001	1.05	0.235	0.196	0.230	1.06	−0.035	0.029	0.228	1.05	0.000	0.022	0.999	1.05
Age	−0.045	0.038	0.238	1.12	−0.049	0.041	0.232	1.14	−0.008	0.012	0.505	1.11	−0.015	0.019	0.430	1.12
Education	0.057	0.048	0.235	1.08	0.067	0.056	0.232	1.09	−0.149*	0.076	0.049	1.07	0.157*	0.080	0.049	1.08
Tenure	0.037	0.031	0.233	1.10	0.035	0.029	0.228	1.11	−0.039	0.033	0.238	1.09	−0.035	0.030	0.244	1.10
*R* ^2^	0.089				0.095				0.083				0.099			
	0.089				0.095				0.083				0.019			
*F*	7.278*		0.001		5.542*		0.001		0.083		0.001		7.535*		0.001	

***p < 0.001, **p < 0.01, *p < 0.05.

Model 3 results show that the square term of illegitimate tasks has a significant positive impact on self-control behavior. It is demonstrated that there is a significant U-shaped relationship between illegitimate tasks and employees’ self-control behaviors, thus verifying H1. Furthermore, following the approach of Haans et al. (2016), combining the results from Model 3, the inflection point of the U-shaped relationship, i.e., the minimum point, is approximately (0.287, −0.088). This result further demonstrates that the U-shaped relationship between illegitimate tasks and self-control behaviors is robust. The results of Model 1 show that the squared term of illegitimate tasks has a significant negative impact on power distance. The results indicate a significant inverted U-shaped relationship between illegitimate tasks and power distance, thereby verifying H2a. Model 4 shows that power distance has a significant negative impact on self-control behavior. H2b has been validated.

This paper employs the MacKinnon et al. (2002) mediation effect test method to verify H2c. First, as shown in Model 3, the square term of non-compliance tasks is significantly positively correlated with the dependent variable, self-control behavior (*β* = 0.579, *p* < 0.01). Second, as shown in Model 1, the squared term of illegitimate tasks is significantly negatively correlated with the mediating variable, power distance (*β* = −1.198, *p* < 0.001). As shown in Model 4, after incorporating power distance, the regression coefficient of the squared term of illegitimate tasks on work self-control behavior is not significant (*β* = 0.409, p > 0.01). The above results indicate that the mediating effect of power distance is substantial, providing preliminary support for H2c. To further verify the mediating effect of power distance, this paper uses the bias-corrected Bootstrap method to resample 5,000 times, yielding estimates of the instantaneous mediating effect at the 95% confidence level. The test results are shown in Table 5. Across different values of the independent variable, the confidence intervals for the mediating effect of power distance do not include 0, indicating that all mediating effects are significant and further verifying H2c.

**Table 5:** Instantaneous indirect effect.

Mediate variable	Dependent variable	Confidence interval		Transient mediation effect
		Lower	Upper	
Power distance	2.611	−0.058	−0.006	−0.025
	3.678	0.009	0.469	0.023
	4.822	0.031	0.071	0.126

**Figure 2:**
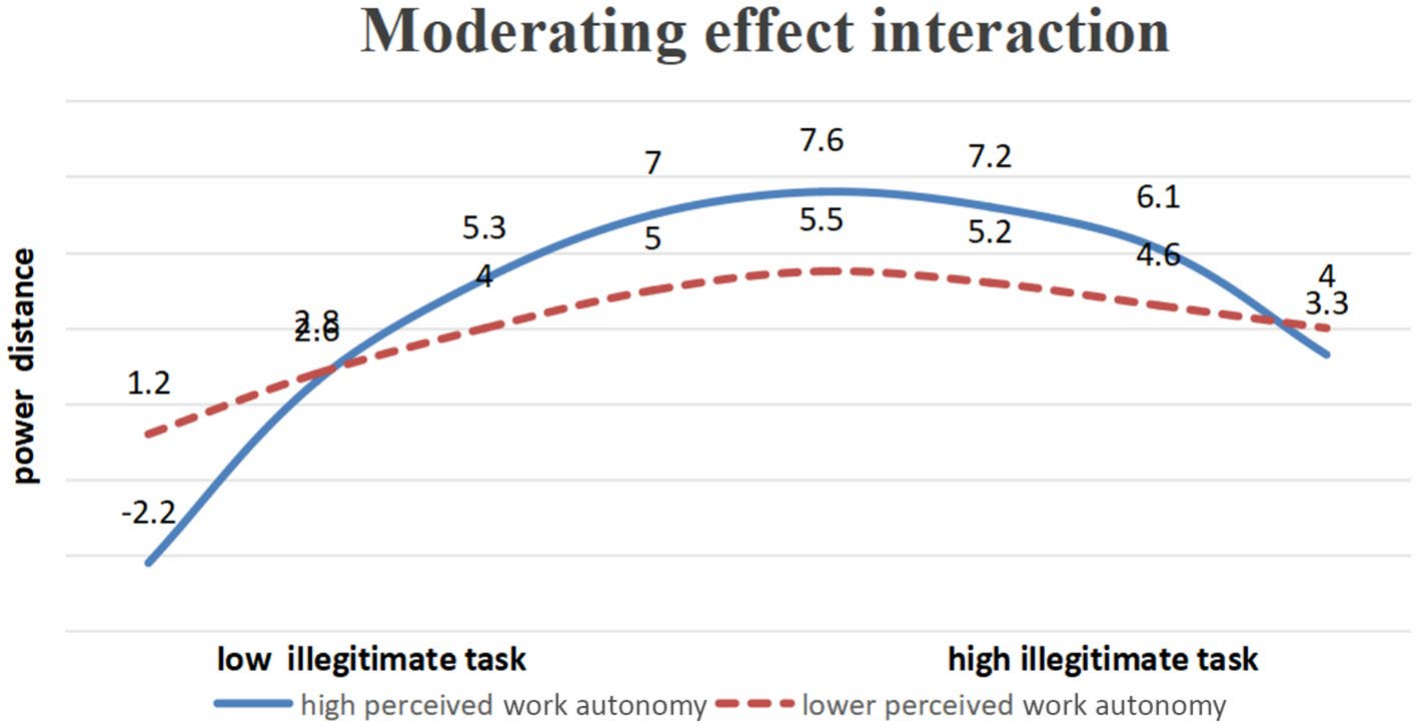
The moderating effect.

Model 2, based on Model 1, added work autonomy perception, the interaction term between illegitimate tasks and work autonomy perception, and the interaction term between the square of illegitimate tasks and work autonomy perception. The results of Model 2 showed that the interaction term between the square of illegitimate tasks and work autonomy perception had a significant negative impact on power distance (*β* = −0.495, *p* < 0.05). It is demonstrated that perceived work autonomy moderates the inverted U-shaped relationship between illegitimate tasks and perceived work autonomy, thus verifying H3. To further analyze the moderating effect of perceived work autonomy, this paper draws a moderation effect diagram. As shown in Figure 2, when employees have a higher perceived level of work autonomy, the inverted U-shaped curve between illegitimate tasks and power distance becomes steeper. In contrast, among employees with lower perceived work autonomy, the inverted U-shaped curve between illegitimate tasks and power distance is flatter. The above results further verify, based on the data points in Figure 2 (illegitimate task levels 0.5–1.0), the exact inflection points of the inverted U-shaped curves for high versus low perceived work autonomy: under high perceived work autonomy, the inflection point is at an illegitimate task level of approximately 0.77 (within the interval [0.7, 1.0]), and the curve rises on [0.5, 0.77] and falls on [0.77, 1.0]; under low perceived work autonomy, the inflection point is approximately 0.78 (within [0.5, 0.9]), with the curve rising on [0.5, 0.78] and falling on [0.78, 1.0]. Both inflection points are close, yet the curve for high autonomy is steeper (larger absolute quadratic coefficient), confirming the moderating role of perceived work autonomy and fully supporting H3.

## 4 Conclusion and discussion

This study is grounded in the conservation of resources (COR) theory and constructs a nonlinear model of how illegitimate tasks influence hotel employees’ self-control behavior. Through empirical analysis of questionnaire data from 536 hotel industry employees, the research hypotheses are verified, leading to the following three main conclusions. First, there is a significant nonlinear relationship between illegitimate tasks and hotel employees’ self-control behavior. As the pressure of illegitimate tasks increases, employees’ self-control behavior first decreases and then gradually increases, exhibiting a U-shaped pattern. Second, power distance plays a significant mediating role in the nonlinear relationship between illegitimate tasks and self-control behavior.

Furthermore, this study further confirms an inverted U-shaped relationship between illegitimate tasks and power distance. Specifically, moderate levels of illegitimate tasks have a positive impact on power distance, whereas excessive pressure from illegitimate tasks brings about an adverse effect on power distance. Finally, this research also verifies the moderating effect of perceived work autonomy in the relationship between illegitimate tasks and power distance. When employees perceive greater work autonomy, the inverted U-shaped relationship between illegitimate tasks and power distance becomes steeper.

### 4.1 Theoretical implication

First, this study extends the literature on illegitimate task research. Most existing studies, grounded in conservation resources (COR) theory, have primarily discussed the negative consequences associated with illegitimate tasks. However, recent research has also begun to verify the potential positive effects of such tasks. Nevertheless, the existing literature generally relies on the assumption of static linear relationships, typically viewing illegitimate tasks solely as negative stressors that hinder performance and examining only their direct or mediating effects. In contrast, this paper adopts a dynamic analytical framework rooted in COR theory. It conducts an in-depth analysis of the double-edged sword effect of illegitimate tasks by examining the interaction between work autonomy and self-control behaviors. The research findings reveal the dynamic relationship between illegitimate tasks and self-control behaviors, thereby integrating the seemingly contradictory findings about the impact of illegitimate tasks reported in the existing literature.

Secondly, this study reveals the underlying mechanism by which illegitimate tasks affect work autonomy. Although existing research has uncovered many negative consequences of illegitimate tasks (i.e., mediator variables), most studies have examined their impact pathways from a linear perspective. This paper reveals that illegitimate tasks exert a nonlinear effect on work autonomy through power distance, analyzing the nonlinear relationship between illegitimate tasks and power distance. This paper breaks through the limitations of simple linear relationships, enriching and improving existing theories. At the same time, existing research on the antecedents of power distance primarily examines the mechanisms underlying the formation of leaders’ and subordinates’ characteristics and behaviors. Still, it ignores the potential impact of work tasks on power distance. The research findings of this paper demonstrate that illegitimate tasks, as a distinct work stressor, can have a nonlinear effect on power distance, thereby expanding the scope of research on antecedent variables of power distance.

Second, this paper deepens the research on the boundary conditions of how illegitimate tasks affect employees. Existing studies categorize the boundary conditions of illegitimate tasks into individual and situational factors, all of which are assumed to follow a linear relationship. This paper introduces perceived work autonomy as a moderating variable and examines its moderating effect on the relationship between illegitimate tasks and employee behavior. Perceived work autonomy, on the one hand, can bring employees a strong sense of control; on the other hand, it can intensify their negative perceptions. Therefore, perceived work autonomy is not a simple buffer or amplifier of the impact of illegitimate tasks; it is a complex moderating factor. When perceived work autonomy is high, the transition from positive effects to adverse effects caused by increased illegitimate tasks occurs more quickly. In contrast, when perceived work autonomy is lower, this transition process is more gradual. The result reveals the complexity of the impact of illegitimate tasks.

Finally, this paper advances research on the impact, effects, and mechanisms of illegitimate tasks. Domestic empirical exploration of illegitimate tasks is in its infancy, and related theoretical research remains insufficient, with specific studies on the hotel industry particularly scarce. Only a few studies have examined the geographic scope of the employee samples, and the existing samples primarily come from business units such as high-end hotels, first-tier cities, catering departments, and front desk departments. Given the service-oriented nature of the hotel industry and its high demands on employees’ emotions, illegitimate tasks are a frequent source of stress for hotel employees. However, moderately challenging tasks can also provide employees with opportunities to acquire new skills, which makes the impact of illegitimate tasks on hotel employees dualistic. Existing literature, due to a lack of in-depth empirical research in the service sector, has an insufficient understanding of the complexity of the impact pathways of illegitimate tasks and has overlooked the nonlinear effects on outcomes such as service deviant behavior. Focusing on hotel industry employees not only helps to improve the theoretical framework of non-compliant task research but also provides industry guidance for the practical management of illegitimate tasks.

### 4.2 Practice implication

The research conclusions contribute to management practices in the hotel industry. First, the research findings indicate a U-shaped, nonlinear relationship between illegitimate tasks and hotel employees’ self-control: appropriate illegitimate tasks reduce self-control, whereas inappropriate ones do not. In contrast, excessively high illegitimate tasks increase it. Therefore, hotel industry managers need to promptly monitor the level of illegitimate tasks and optimize their allocation so that hotel employees undertake an appropriate number of legitimate tasks. Additionally, hotel organizations can enhance employees’ autonomy to address challenges posed by illegitimate tasks. Specifically, hotel service flexibility can be improved by granting hotel employees greater decision-making autonomy. Additionally, through systematic training and career counseling, employees’ knowledge reserves, professional skills, and psychological qualities can be enriched, thereby increasing their confidence and sense of self-efficacy in handling illegitimate tasks.

Secondly, research indicates that power distance relationships significantly moderate the relationship between illegitimate tasks and hotel employees’ self-control behavior. This conclusion suggests that power distance serves as an important moderating factor in the relationship between illegitimate tasks and hotel employees’ self-control. Hotel managers can effectively mitigate the adverse effects of illegitimate tasks by providing sufficient psychological support and resource guarantees to hotel employees who undertake them. Specifically, a comprehensive employee care system can be established to provide timely emotional guidance. At the same time, organizations should offer professional psychological counseling services. When employees face pressure from illegitimate tasks, effective maintenance of their mental health can be achieved through methods such as individual counseling, group counseling, or stress management.

Finally, this study found that perceived work autonomy moderates the impact of illegitimate tasks on hotel employees. Based on this, managers should implement appropriate guidance mechanisms for employees with higher perceived work autonomy, promptly attend to their psychological states, and foster positive interactions with the organization. Specifically, for employees with higher perceived work autonomy, managers can introduce a “task adjustment request” form that allows them to propose modifications (e.g., deadline extension, resource reallocation, partial delegation) when a task is perceived as illegitimate; a weekly 10-min autonomy check- in where managers actively ask about task meaningfulness and offer cognitive reappraisal guidance (e.g., reframing the task as a learning opportunity); and a self-control recovery micro-training (e.g., a 5-min guided breathing or perspective-taking exercise before and after executing a depleting task). For employees with lower perceived work autonomy, who often lack initiative and struggle to respond effectively, managers should provide necessary skill training and support resources to minimize the negative impact of illegitimate tasks. Concretely, this includes a three-item illegitimate task filtering checklist (“Does this task clearly belong to my role?” “Do I have the required resources?” “Can I seek help without negative consequences?”); a mandatory task clarification meeting triggered when two criteria are unmet; a 90-min negotiation skills training module with role-play using a simple three-step script (“Acknowledge-State limitation-Propose alternative”); and a digital or physical “resource map” displaying whom to contact for each type of task difficulty (e.g., technical support, extra staffing, priority override). By replacing vague guidance with these concrete task constraints and modular training practices, managers can more effectively buffer the negative impact of illegitimate tasks on hotel employees’ control behavior (Rodriguez et al., 2026).

### 4.3 Research limitations and future prospects

*Firstly*. Cultural limitations and external validity concerns.

This study was conducted exclusively within the Chinese context—a setting characterized by a large population, high deference to authority, and a tendency among employees to rarely question illegitimate tasks openly. This cultural specificity may either amplify or suppress the relationships observed. As a result, the findings may not be directly generalizable to low-power-distance or more egalitarian cultural contexts.

*Secondly*. Bias risks associated with single-source data. Although a two-wave data collection design was employed to mitigate common-method bias, all variables—including illegitimate tasks, power distance, work autonomy, self-control behavior, and demographics—were still measured via employee self-reports. Self-reported data are inherently susceptible to recall bias, social desirability bias, and consistency motives.

*Thirty*. Potential distortion from unmeasured individual traits. Even with temporal separation between the two waves, the fact that both independent and dependent variables originated from the same source means that unmeasured individual dispositional factors (e.g., negative affectivity, trait self-control) may still inflate or distort the observed relationships.

*Fourth*. Correlational interpretation only; causal inference not possible. This study cannot establish definitive causal relationships. The observed associations should be interpreted as correlational rather than strictly causal. The proposed U-shaped or inverted U-shaped relationships require further validation using multi-source designs (e.g., supervisor-rated self-control behavior) or experimental approaches that better control for confounding variables and reverse causality.

## Data Availability

The raw data supporting the conclusions of this article will be made available by the authors, without undue reservation.
