# Correction: The double-edged sword effect of illegitimate tasks on hotel industry employees' self-control behavior

**DOI:** 10.3389/fpsyg.2026.1879261

**Published:** 2026-05-26

**Authors:** Wenjing Ke, Bingjie Sun

**Affiliations:** 1 College of Business, Wuyi University, Wuyishan, China; 2 School of Business Administration, Xinjiang University of Science & Technology, Korla, China

**Keywords:** double-edged sword effect, employee self-control behavior, illegitimate tasks, power distance, work autonomy

An incorrect **Funding** statement was provided. The funding statement for Grant No. 2026-NJ-ZD02 incorrectly identified the funding institution. The correct funder of Grant No. 2026-NJ-ZD02 is the Southern Xinjiang Digital Economy and High-Quality Development Research Center, Xinjiang Philosophy and Social Sciences Innovation Platform. The correct **Funding** statement reads:

“The author(s) declared that financial support was received for this work and/or its publication. This research was funded by the Scientific Research Start-up Project for Introduced Talents of Wuyi University (Grant No. YJ202504) awarded to Wenjing Ke, and the Investigation on Digital Transformation of Characteristic Forest and Fruit Industry in Southern Xinjiang (Grant No. 2026-NJ-ZD02), supported by the Southern Xinjiang Digital Economy and High-Quality Development Research Center, Xinjiang Philosophy and Social Sciences Innovation Platform, awarded to Bingjie Sun. The funders had no role in the study design, data collection and analysis, decision to publish, or preparation of the manuscript.”

The original version of this article has been updated.

